# Statistical invisibility and surgical harm: gossypiboma and the hidden safety crisis for women in Pakistan

**DOI:** 10.1097/MS9.0000000000005068

**Published:** 2026-04-27

**Authors:** Zahabia Adnan, Amna Kashif, Ahmed Asad Raza, Abedin Samadi

**Affiliations:** a Department of Medicine, Jinnah Sindh Medical University, Karachi, Pakistan; b Department of Medicine, Kabul University of Medical Sciences, Abu Ali Ibn Sina, Kabul, Afghanistan

Gossypiboma is a medical mishap that refers to the retention of surgical sponges and gauze after a surgical procedure, commonly due to human error that result in severe consequences. Although considered rare, gossypiboma remains an under-recognized driver of maternal harm, disproportionately reported after gynecologic and obstetric surgeries, highlighting a serious statistical invisibility that directly undermines women’s patient safety. In a recent case report, exploratory laparotomy revealed a retained gauze in a woman who showed the symptoms of acute abdominal obstruction merely within a week of cesarean section^[^1^]^. Such events can be entirely preventable under appropriate surgical protocols. Hence, bridging this gap is not optional; it is a constitutional obligation. Patient safety cannot depend on concealment or chance.

The consequences of retained surgical items (RSIs) in Pakistan require immediate action due to clinical safety issues, societal impact, and disparities in patient care. As it is reported in the national survey that cesarean section rates in Pakistan have dramatically increased from 3% in 1990 to 20% in 2018, this rise has significantly increased women’s vulnerabilities to surgical risks, including gossypiboma^[^2^]^. This sharp rise is due to the profit-driven practices in private hospitals, as C-sections are quicker and generate high revenues. Under-reporting of such cases is particularly driven by the fear of legal repercussions, anxiety, social stigma to pursue postpartum, and weak organizational accountability, which ultimately exacerbate the injury and result in gendered patient-safety failures. Postoperative symptoms are relegated to “normal recovery,” causing diagnostic delay. Moreover, such events are commonly associated with financial burdens characterized by extended hospitalizations, repeated surgeries, and potential litigation. This results in the patient avoiding the post-surgical procedures, as it pushes already vulnerable households deeper into poverty.

High pressure and emergency situations often cause inconsistent sponge and instrument count in operation theaters, a problem especially amplified in lower-middle-income countries like Pakistan, where tertiary care hospitals are usually understaffed and doctors are overworked, with 46% reported to be burned out^[^3^]^. In situations like these, the chance of missed items rises sharply, particularly during prolonged or emergency procedures. In addition to this, lack of resource constraints, with most hospitals not having access to safeguards, such as radio-opaque sponges or reliable intra-operative imaging can further increase the risk of error. As a part of the system, many medical facilities conceal cases of RSIs to avoid lawsuits or reputational loss, and poor medicolegal frameworks provide little support for open reporting. Such concealment also contradicts Pakistan’s constitutional guarantees under Article 9 of the Constitution, the right to life extends to health and medical safety, as affirmed by the Supreme Court in *Shehla Zia v. WAPDA (PLD 1994 SC 693)*^[^4^]^. RSIs, therefore, are not only a clinical error but a violation of fundamental rights. This lack of accountability breeds a punishing culture in which errors are hidden rather than addressed, which leads to compromised patient safety.

Preventing gossypiboma, while challenging, it is achievable with reforms tailored to Pakistan’s realities. The foundation must be strictly enforced in accordance with the WHO Surgical Safety Checklist, supported by regular audits and the use of WHOBARS to strengthen team communication^[^5^]^. Another course of action should be the mandation of the “hard stop” protocol before closure, which combines a final count with a surgeon-led cavity sweep, as well as clearly named staff responsible for verification. In low-to-moderate income country hospitals, assigning explicit accountability for sponge and instrument counts is a practical and cost-effective solution. While advanced tools such as radio frequency identification or bar-coded sponges and intra-operative detection wands have been shown to reduce errors, they remain financially unrealistic for most facilities in Pakistan^[^6^]^. In the interim, trigger-based imaging with X-ray or ultrasound for discrepant counts or emergency conversions is a viable alternative. Outside of the operating room, a nationwide non-punitive registry, open reporting, and patient-centered permission to disclose the risk of RSI are required^[^7^]^. Open disclosure, dismissals, and rapid remedial care must replace concealment, transforming the culture from blame to accountability and learning.

Gossypiboma is not merely a surgical mishap, but rather a system-wide and gendered patient-safety failure that continues to undermine women’s health in Pakistan. The frameworks exist, including constitutional protections, ethical codes, and international safety checklists, but they are not followed. This is the time to act ensuring that no patient should leave a surgery with a foreign body inside her, in a place that is meant to heal her.

To ensure methodological transparency and ethical compliance in manuscript preparation, we adhered to the recently developed TITAN (Transparency in the Reporting of Artificial Intelligence) 2025 guidelines, which outline standards for responsible AI use in biomedical writing and publishing^[^8^]^.

## Data Availability

No datasets were generated or analyzed during the current study.
